# The effects of major abdominal surgery on skeletal muscle mitochondrial respiration in relation to systemic redox status and cardiopulmonary fitness

**DOI:** 10.3389/ebm.2025.10254

**Published:** 2025-02-21

**Authors:** Jia L. Stevens, Helen T. McKenna, Magdalena Minnion, Andrew J. Murray, Martin Feelisch, Daniel S. Martin

**Affiliations:** ^1^ Department of Anaesthesia, Royal Sussex County Hospital, University Hospital Sussex NHS Foundation Trust, Brighton and Hove, United Kingdom; ^2^ Division of Surgery and Interventional Science, Faculty of Medical Sciences University College London, London, United Kingdom; ^3^ Peninsula Medical School, Faculty of Medicine and Dentistry, University of Plymouth, Plymouth, United Kingdom; ^4^ Clinical and Experimental Sciences and Integrative Physiology and Critical Illness Group, Faculty of Medicine, Southampton General Hospital, University of Southampton, Southampton, United Kingdom; ^5^ Department of Physiology, Development and Neuroscience, Faculty of Biology, School of Biological Sciences, University of Cambridge, Cambridge, United Kingdom

**Keywords:** cardiopulmonary, mitochondrial respiration, redox, antioxidants, perioperative

## Abstract

More complex surgeries are being performed in increasingly sicker patients, resulting in a greater burden of postoperative morbidity. Delineating the metabolic and bioenergetic changes that occur in response to surgical stress may further our understanding about how humans respond to injury and aid the identification of resilient and frail phenotypes. Skeletal muscle biopsies were taken from patients undergoing hepato-pancreatico-biliary surgery at the beginning and end of the procedure to measure mitochondrial respiration and thiol status. Blood samples were taken at the same timepoints to measure markers of inflammation and systemic redox state. A sub-group of patients underwent cardiopulmonary exercise testing prior to surgery, and were assigned to two groups according to their oxygen consumption at anaerobic threshold (≤10 and >10 mL/kg/min) to determine whether redox phenotype was related to cardiorespiratory fitness. No change in mitochondrial oxidative phosphorylation capacity was detected. However, a 26.7% increase in LEAK (uncoupled) respiration was seen after surgery (P = 0.03). Free skeletal muscle cysteine also increased 27.0% (P = 0.003), while S-glutathionylation and other sulfur and nitrogen-based metabolite concentrations remained unchanged. The increase in LEAK was 200% greater in fit patients (P = 0.004). Baseline plasma inflammatory markers, including TNF-⍺ and IL-6 were greater in unfit patients, 96.6% (P = 0.04) and 111.0% (P = 0.02) respectively, with a 58.7% lower skeletal muscle nitrite compared to fit patients. These data suggest that oxidative phosphorylation is preserved during the acute intraoperative period. Increase in free cysteine may demonstrate the muscle’s response to surgical stress to maintain redox balance. The differences in tissue metabolism between fitness groups suggests underlying metabolic phenotypes of frail and resilient patients. For example, increased LEAK in fitter patients may indicate mitochondrial adaptation to stress. Higher baseline measurements of inflammation and lower tissue nitrite in unfit patients, may reflect a state of frailty and susceptibility to postoperative demise.

## Impact statement

Improved access to surgery has increased the global burden of postoperative pathology. Understanding the mechanisms that drive postoperative demise, and identifying at-risk patients are paramount to the advance of perioperative medicine. This study provides new insight into the body’s responses to acute surgical stress, demonstrating that the initial response to injury does not solely release markers of cell/tissue damage, but also markers of adaptation, with evidence of mitochondrial bioenergetic alterations and the maintenance of sulfur and nitrogen-based metabolites. Our study also provides phenotypic profiles of patients representing perioperative resilience and frailty. The association of reduced baseline aerobic capacity with increased levels of cyclic guanosine monophosphate, inflammation, and intraoperative mitochondrial uncoupling, is indicative of a biochemical phenotype for deconditioned and frail patients. The identification of such responses to major surgery and their variability brings us closer to personalised and stratified medicine.

## Introduction

As surgical technology advances, we are performing more complex procedures in frailer and more multi-morbid patients than ever before, and the consequences of undergoing surgery can be life changing. Whilst the intention, particularly in cancer surgery, is to provide a cure, complications can occur, resulting in a proportion of these patients being left with deficits for months or years to come. Characterising the metabolic and bioenergetic changes that occur after acute surgical stress may further our understanding of how the human body responds to injury and how these responses differ between individuals. Identifying a physiological and biochemical phenotype that characterises resilience and protection is an important step for making progress in perioperative medicine.

The pivotal role that mitochondria play in cellular bioenergetics is well established, but their role in redox metabolism, including the production and scavenging of reactive oxygen species (ROS) and reactive nitrogen species (RNS) which can promote a state of oxidative and/or nitrosative stress, is being recognised only more recently. In addition, reactive sulfur species (e.g., hydrogen sulfide and per/polysulfides) and sulfur-containing compounds such as thiols have potent antioxidant capacity and can produce a wide array of oxidation products. When combined, these redox metabolic reactions can be conceptualised using the “reactive species interactome” framework [1].

Altered mitochondrial function, in conjunction with oxidative stress, has been linked to the pathogenesis of multiple chronic conditions and to the mechanisms that underlie frailty and physical fitness [2]. Physical fitness is associated with many health benefits, including reductions in occurrence of metabolic and cardiovascular diseases and cancer [3], increased longevity and a reduction in the development of age-related illnesses [4, 5]. Cardiopulmonary exercise testing (CPET) provides a direct measure of cardiorespiratory fitness where oxygen consumption (VO_2_) and carbon dioxide production (VCO_2_) in response to increasing levels of physical activity provide an indirect yet integrative measure of tissue respiration and hence mitochondrial function. Data from CPETs are used as predictor of a patient’s physiological response to the stress of surgery. Lower levels of preoperative aerobic fitness [peak VO_2_ and VO_2_ at anaerobic threshold (AT)] have repeatedly been associated with postoperative morbidity [6, 7].

The contributions of altered mitochondrial respiratory function with oxidative stress in an acute surgical setting are less well established and described. They have been reported in one animal study and one human study (of skeletal muscle biopsies) to date [8, 9]. Both studies observed markers of altered mitochondrial respiration after surgery. In addition, greater release of ROS was associated with a loss of mitochondrial membrane potential [10]. In the perioperative setting, an AT of <11 mL/kg/min, has been associated with increased levels of preoperative markers of inflammation [11]. Peak VO_2_ levels have been positively correlated with mitochondrial respiratory capacity, and in particular of oxidative phosphorylation [12]. However, the links between fitness, redox metabolism, mitochondrial respiration and the effects of surgery have not yet been addressed.

We conducted a single-centre, prospective exploratory study of patients undergoing major hepato-pancreatico-biliary (HPB) surgery at the Royal Free Hospital (London, UK). Results from the main study have been previously reported [13]. A subgroup of patients from the main study underwent additional investigations. We hypothesized that less fit patients would display a greater degree of perturbation to whole-body redox balance with greater systemic oxidative stress and early changes in mitochondrial respiratory function in skeletal muscle following surgery. Specifically, we were interested to learn: 1) how major abdominal surgery affects mitochondrial respiration and redox status of skeletal muscle under conditions of systemically increased oxidative stress, and 2) whether those changes differ between fit and unfit individuals.

## Materials and methods

### Participants

Patients for this sub-study were selected from a larger study of the effects of major abdominal surgery on circulating redox markers [13]. All patients were approached up to 1 month prior to scheduled surgery to seek their agreement to participate. Inclusion criteria included: major (intra-cavity) inpatient surgery; age ≥18 years; planned general anaesthesia; calculated morbidity risk ≥40% (Portsmouth Physiological and Operative Severity Score for the enUmeration of Mortality and morbidity – P-POSSUM). Exclusion criteria included: mitochondrial disease; emergency surgery; lack of capacity; prisoners. In addition, patients in the sub-study were required to undergo skeletal muscle biopsies at the beginning and end of surgery and (if feasible) a cardiopulmonary exercise testing prior to their surgery (see Supplementary Figure S1 *CONSORT diagram*).

The study was designed and reported according to the Strengthening the Reporting of Observational Studies in Epidemiology (STROBE) Guidelines [14]. Ethical approval was obtained from the West London Research Ethics Committee and Human Research Authority [214019]. All patients provided written informed consent prior to surgery.

### Cardiopulmonary exercise testing

CPET took place on a cycle ergometer according to a local protocol, which followed the perioperative CPET consensus clinical guidelines [15]. Exercise was conducted on an electromagnetically braked cycle ergometer (Lode BV medical technology), following a protocol of: 3 minutes rest; 2 minutes of freewheel pedalling; ramped incremental cycling until the patient could no longer continue; and a 5-min recovery period of freewheel pedalling. Ventilation and gas exchange were measured through use of a metabolic cart (Metalyzer 3B, Cortex Biophysics GmbH) and the data was analysed using the Metasoft 3.9 software. The ramp gradient was set to 10–25 W/min based on the participant’s self-reported level of activity. Resting flow-volume loops were used to derive measures of forced expiratory volume over one second. AT was estimated in the conventional manner, involving the use of the three-point discrimination technique (identification of excess VCO_2_ relative to VO_2_; identification of hyperventilation relative to oxygen; hyperventilation excluded relative to carbon dioxide). Two separate reviewers determined the AT values independently and the average of the measurements was taken as the final AT. When there was disagreement of >10%, a third reviewer was invited to be the final adjudicator, and the average of the two measurements within 10% was taken as the final AT. Peak VO_2_ was averaged over the last 30 s of exercise.

### Anaesthetic and surgical techniques

For details of anaesthesic and surgical techniques the reader is referred to the methodology section of the main study published elsewhere [13] and the supplementary section of the present paper.

### Blood collection

Samples of venous blood were collected at baseline (after induction of anaesthesia but prior to the first surgical incision) and at the end of surgery (EoS) after wound closure. Samples were immediately placed on ice and centrifuged at 2000 × *g* for 15 min at 4°C. Plasma and serum samples were divided into aliquots for storage at −80°C.

### Blood analysis

The following markers were chosen on the basis of their relationship within the “reactive species interactome” framework to include lipid oxidation products such as malondialdehyde (MDA), 4-hydroxynonenal (4-HNE) and 8-iso-prostaglandin F_2⍺_ (8-isoprostanes); markers of total reducing capacity (TRC), total free thiols (TFTs) and ferric reducing ability of plasma (FRAP); as well as markers of NO production, metabolism and availability including cyclic guanosine monophosphate (cGMP), nitrite, nitrate and total nitroso-species (RXNO). Interleukin-6 (IL-6) and tumour necrosis factor alpha (TNF-⍺) were also measured to evaluate inflammation. Pristine (first thaw from −80°C) serum samples were used throughout; for details of method of analysis please refer to reference 13^13^ and the Supplementary Material.

### Skeletal muscle biopsy

Vastus lateralis muscle was biopsied at two separate timepoints under general anaesthesia, which was coupled with blood sample extraction; at baseline and EoS, using previously described methods [16]. Biopsies were taken from the mid-thigh using Tilley-Henckel forceps under local anaesthesia (1% lidocaine) of the skin and superficial muscle fascia. A 5 mm incision was made, and 100 mg wet-weight tissue was collected. The sample was divided, with 50 mg allocated for immediate respirometric analysis and the remainder snap frozen in liquid nitrogen and stored at −80°C until later analysis. The muscle sample was divided into aliquots, the sample for high-resolution respirometry (HRR) was immediately placed in ice-cold biopsy preservation medium (BIOPS): [CaK_2_EGTA (2.77 mM), K_2_EGTA (7.23 mM), MgCl_2_.6H_2_O (6.56 mM), taurine (20 mM), PCr (15 mM), imidazole (20 mM), DTT (0.5 mM), MES (50 mM) and Na_2_ATP (5.77 mM) at pH 7.10], which was filtered and stored at −40°C until use to prevent bacterial growth. The aliquots for muscle metabolomics were snap frozen in liquid nitrogen and subsequently stored at −80°C for later analysis.

### Sample preparation and high resolution respirometry

Skeletal muscle fibre bundles were prepared from the respirometry-designated sample according to previously described methods [17]. After permeabilisation of the sarcolemmal membrane using saponin (50 μg/mL, in ice cold BIOPS, rocked for 20 min at 20 rpm), fibre bundles were rinsed in respiration medium (MiR05, outlined below) blotted on filter paper and weighed using a microbalance (Mettler-Toledo). Respiration of fibre bundles was then measured in mitochondrial respiration medium (MiR05) containing EGTA (0.5 mM), MgCl_2_.6H_2_O (3 mM), K-lactobionate (60 mM), taurine (20 mM), KH_2_PO_4_ (10 mM), HEPES (20 mM), sucrose (110 mM) and defatted BSA (1g.L-1) at pH 7.4, using the substrate-uncoupler-inhibitor titration (SUIT) protocol described below. All assays were performed, in duplicate, using an Oxygraph O2K (Oroboros Instruments, Innsbruck), at 37°C with oxygen concentrations kept between 250 and 400 µM and constant stirring to prevent diffusion limitation of respiration. Respirometry was performed by the same operator throughout the study.

Oxygen consumption of permeabilised muscle fibres were measured using a fatty acid oxidation (FAO)-mediated SUIT protocol. In brief, mitochondrial respiratory states were recorded following stepwise titrations. Addition of malate and octanoylcarnitine supported LEAK respiration (LEAK_FAO_), with oxygen consumption not coupled to oxidative phosphorylation (OXPHOS); addition of adenosine diphosphate (ADP) resulted in OXPHOS supported by fatty acid oxidation (FAO_OXPHOS_); addition of pyruvate reconstituted the Krebs cycle (MOP_OXPHOS_), and glutamate produced OXPHOS supported by complex I (CI_OXPHOS_). Addition of succinate stimulated OXPHOS supported by complexes I and II, with a further titration of ADP to achieve maximum complex I and II-mediated OXPHOS (MAX OXPHOS). Titration of the protonophore, carbonyl cyanide p-trifluoro-methoxyphenyl hydrazone (FCCP), resulted in maximal electron transfer system (ETS) capacity, unlimited by the phosphorylation system (CI + II_*ETS*_). The relative contribution of complex II was assessed by addition of the complex I inhibitor, rotenone (CII_*ETS*_). For further information refer to Supplementary Table S1.

### Metabolomic analysis of skeletal muscle

Muscle biopsies were accurately weighed and mixed with 300 µL of homogenisation buffer (10 mM phosphate-buffered saline with 10 mM N-ethylmaleimide (NEM) and 2.5 mM EDTA) and homogenised by 8 up-and-down strokes under ice-cooling using a Kimble all-glass tissue grinder attached to a GlasCol GT Series stirrer. Tissue homogenates were then split and treated depending on the markers to be analysed, as detailed below. One 100 µL aliquot of the muscle homogenate was deproteinised by precipitation with ice-cold methanol (1:1, v:v) and centrifugation at 16,000 × *g* for 20 min. Clear supernatants were analysed for nitrite (NO_2_^−^) and nitrate (NO_3_^−^) using a dedicated high-performance liquid chromatography system for NOx analysis (ENO-30 with AS-700 autosampler, Eicom/Amuza), and data were processed using the Clarity software. The remainder of the muscle homogenates was utilised to evaluate thiol redox status using an ultra-high performance liquid chromatography tandem mass spectrometry (UPLC-MS/MS) method described in detail elsewhere [18]. A 100 µL supernatant of the muscle homogenates was mixed 1:1 with internal standards before centrifugation and injection onto the LC-MS/MS system (Aquity/XEVO-TQS, Waters). The method was used to separate and quantify biological aminothiols such as reduced and oxidized glutathione (GSH, GSSG), cysteine (Cys/cystine) and homocysteine (HCys/homocystine) as well as sulfide (HS^−^). In addition to the free thiols, total thiol concentrations (free + protein-bound forms and disulfides) were determined after sample pre-processing with dithiothreitol (DTT). For this purpose, an additional 50 µL aliquot of muscle homogenate was subjected to reduction by DTT (50 mM, 1:1, v:v) followed by incubation for 30 min at room temperature before addition of 400 µL of 100 mM NEM for derivatization of liberated thiols. After 15 min incubation at room temperature, derivatized samples were spiked with internal standards, subjected to ultrafiltration for protein removal and injected onto the LC-MS/MS system.

### Clinical data collection and sample size determination

Clinical data were collected prospectively throughout the perioperative admission and entered into a database.

### Statistical analysis

Data were assessed for normality by visual examination of histograms and using the Shapiro-Wilk test. Data were presented as median and interquartile range (IQR). Wilcoxon signed-rank sum was used for paired tests and Mann-Whitney U for two independent samples for non-normally distributed data. Changes in intraoperative concentrations of metabolic indices (calculated as EoS concentration – baseline concentration) was used to reflect intraoperative trajectory/dynamics of the metabolic profile. AT was chosen as a discriminatory measure of cardiopulmonary fitness. A cut-off point of 10 mL/kg/min was chosen to dichotomise the patients into two groups, labelled “fit” and “unfit.” This threshold was chosen because it was found to be specifically associated with outcomes after HPB surgery [6]. The linear relationships between non-normally distributed continuous data were assessed using Pearson’s correlation. Missing values were excluded from the analysis. All tests were two-tailed and P < 0.05 was selected as the threshold for statistical significance. In view of the exploratory nature of the study, a decision was made not to correct for multiplicity. Whilst this increased the risk of generation of a type-1 error, it simultaneously reduced the risk of generation of a type-2 error, which was considered important in work of this nature. Statistical analyses were carried out using IBM SPSS version 26 software and graphs were created using GraphPad Prism 8 software.

## Results

### Clinical data

37 patients underwent paired (baseline and EoS*)* muscle biopsies, 33 sets were used for HRR and 37 sets of samples were used for metabolomic analyses. A sub-group of 23 patients underwent CPET prior to surgery.

Patient demographics and baseline preoperative data can be found in Table 1. For detailed intraoperative and postoperative clinical data refer to Supplementary Tables S2, S3. Of the 37 patients, 34.3% had pancreatic surgeries, 45.7% hepatic resections, and 20.0% palliative procedures. CPET measurements are summarised in Table 2. Participants had a median AT of 11.5 mL/kg/min and VO_2_ peak of 16.5 mL/kg/min. The median number of days on ICU and in hospital were 2.7 (1–2.8) and 9.0 days (6.2–14.8) respectively.

**TABLE 1:** Baseline patient data. Baseline patient demographics and co-morbidities with absolute numbers and percentages, unless otherwise stated and presented as median and IQR.

Characteristics	
Age (years)	67.0 (58.0–69.5) Median (IQR)
Gender (male: female)	24:10 (70.6:29.4)
BMI (kg/m^2^)	24.3 (22.7–28.6) Median (IQR)
Ethnicity [n (%)]	
White	32 (94.1)
Asian	1 (2.9)
Black	1 (2.9)
Smoking status [n (%)]	
Yes	4 (11.8)
No	19 (55.9)
Ex-smoker	11 (32.4)
Alcohol [n (%)]	
Yes	11 (32.4)
No	23 (67.6)
Consumption (units per week)	2 (2–6.5)
ASA [n (%)]	
I	3 (8.8)
II	19 (58.8)
III	11 (32.4)
IV	0 (0.0)
Comorbidities [n (%)]	
Cardiovascular	19 (55.9)
Hypertension	12 (35.3)
Ischaemic heart disease	4 (11.8)
Heart failure	0 (0.0)
Arrythmia	3 (8.8)
Valvular heart disease	0 (0.0)
Cerebral vascular disease	1 (2.9)
Peripheral vascular disease	2 (5.9)
Respiratory	6 (17.6)
COPD	3 (8.8)
Asthma	2 (5.9)
OSA	1 (2.9)
Other	0 (0.0)
Endocrine and metabolic	16 (44.4)
Diabetes	9 (26.2)
Hypercholesterolaemia	5 (14.7)
Other	2 (5.9)
Renal disease	0 (0.0)
Rheumatological	4 (11.8)
Other systemic disease	2 (5.9)
Diagnosis [n (%)]	
Pancreatic cancer	12 (35.3)
Liver metastasis	10 (29.4)
Cholangiocarcinoma	5 (14.7)
Hepatocellular carcinoma	4 (11.8)
Neuroendocrine tumour	1 (2.9)
Ampulla and duodenal cancer	1 (2.9)
Other	1 (2.9)
Neo-adjuvant chemoradiotherapy within the last year [n (%)]	7 (20.6)

**TABLE 2:** Baseline CPET data. CPET data of the main test group, and divided into unfit and fit groups based on AT (cut-off ≤10 mL/kg/min).

Fitness indices derived from CPET	Total Median (IQR)	Unfit (n = 6) Median (IQR)	Fit (n = 17) Median (IQR)
AT (mL/kg/min)	11.5 (4.3)	9.0 (2.0)	13.5 (3.5)
VO_2_ peak (mL/kg/min)	16.5 (8.0)	15 (1.5)	22.9 (9.0)
VE/VCO_2_	29.0 (4.3)	31.4 (6.9)	27.9 (3.4)
Peak HR (bpm)	131 (39)	142 (22)	125 (21)
O_2_ pulse at peak VO_2_ (mL)	10.5 (6.5)	9 (1.9)	11.6 (6.60)
Work ramp rate (W)	15 (5)	20 (5)	15 (5)
Work rate at peak VO_2_ (W/min)	115.0 (94)	105 (31)	126 (69)

### Changes in skeletal muscle mitochondrial respiratory capacity and redox metabolome between baseline and end of surgery

**FIGURE 1:**
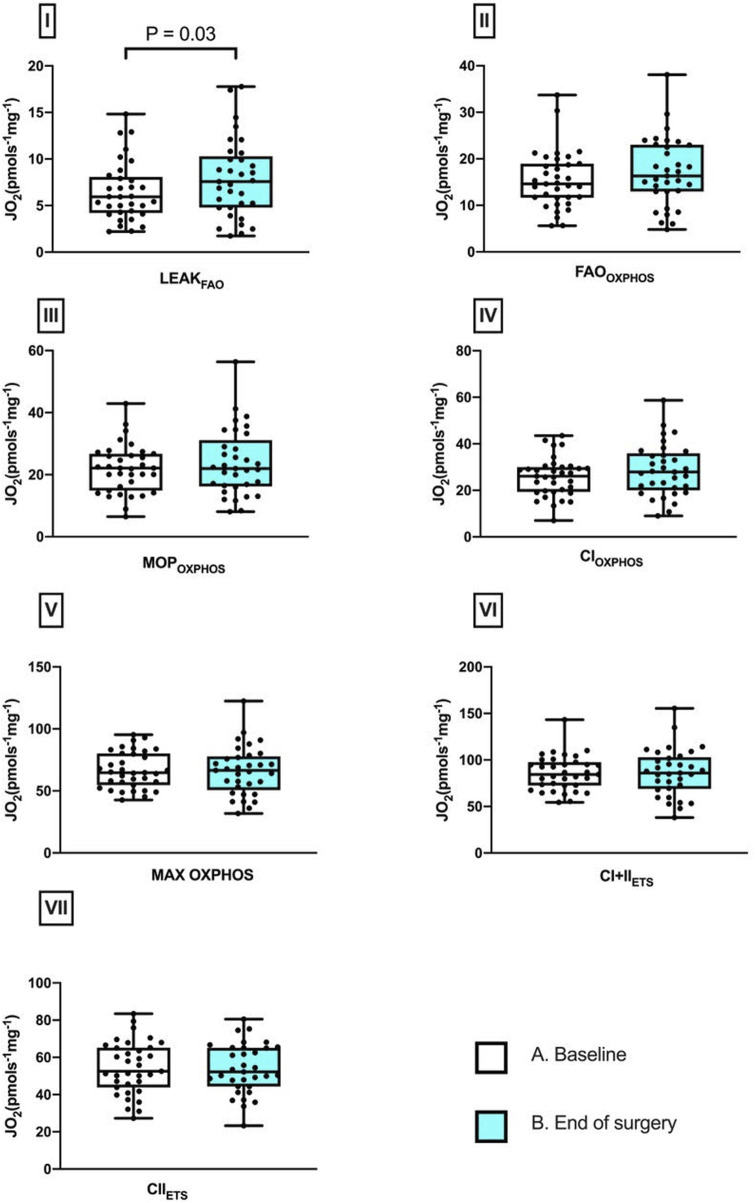
Perioperative changes in skeletal muscle mitochondrial respiratory states. Differences between baseline and after surgery in I, LEAK_FAO_; II, FAO_OXPHOS_; III, MOP_OXPHOS_; IV, CI_OXPHOS_; V, CI + II_OXPHOS_; VI, CI + II_ETS_; and VII, CII_ETS_ (median and IQR). Pairwise comparisons were performed using the Wilcoxon signed ranks test (n = 33).

**FIGURE 2:**
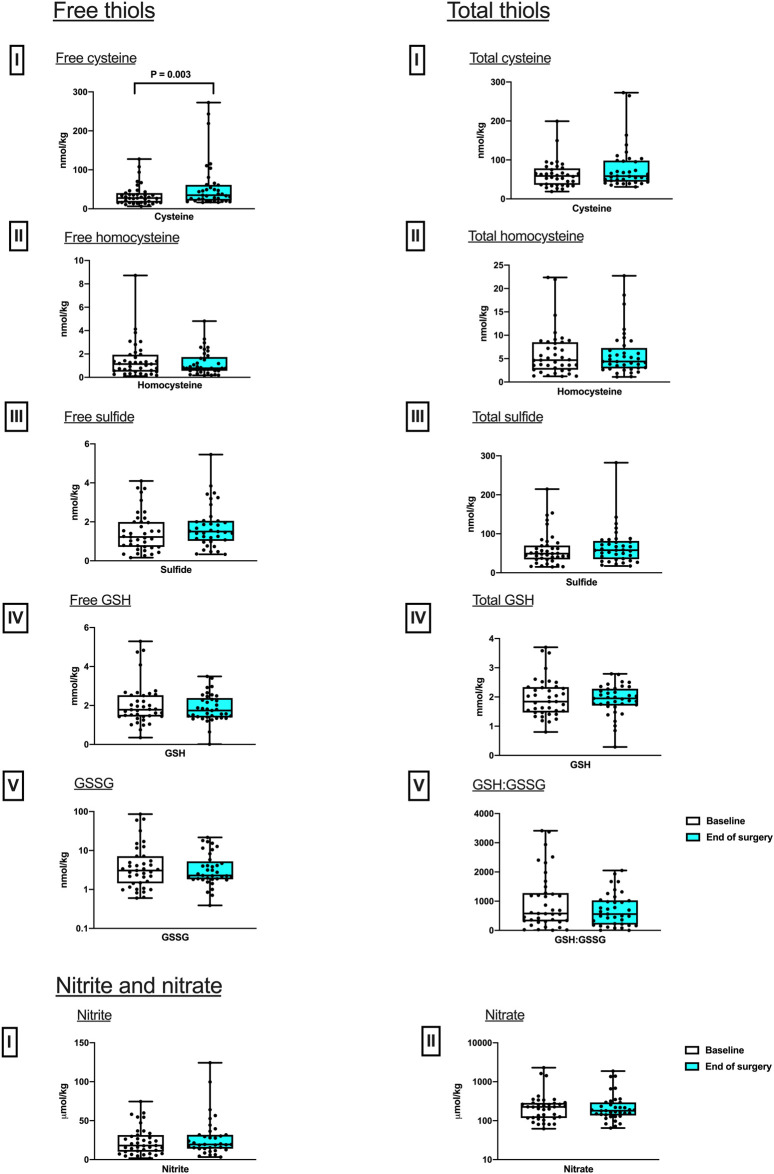
Perioperative changes in skeletal muscle sulfur (free and total thiols) and nitrogen-based metabolites. Differences in concentrations between baseline and after surgery in Free, I, Cysteine; II Homocysteine; III, Sulfide; IV, GSH; and V, GSSG; and total, I, Cysteine; II Homocysteine; III, Sulfide; IV, GSH; as well as V, the ratio of reduced over oxidized glutathione, GSH:GSSG. Nitrite and nitrate, I, Nitrite; II Nitrate. (median and IQR). Pairwise comparisons were performed using the Wilcoxon signed ranks test (n = 37).

Skeletal muscle respiratory function measured by HRR revealed a 26.7% increase in LEAK_**FAO**_ respiration from baseline to EoS (P = 0.03). No difference in any other measured respiratory state, including maximal oxidative phosphorylation capacity, was detected between these two timepoints (Figure 1). While the intramuscular concentrations of nitrite and nitrate as well as those of free and total thiols (aminothiols and sulfide) did not differ between baseline and end of surgery, a 27.0% increase in free cysteine content was observed after surgery (Figure 2).

### Perioperative differences in blood and skeletal muscle markers based on patients’ cardiorespiratory fitness

Baseline levels of circulating antioxidant, lipid oxidation, NO and inflammation status were compared in the fit and unfit patient groups. No differences between the groups were detected in serum markers of antioxidant capacity (TRC) or lipid oxidation. Plasma cGMP was found to be 81.6% higher in the unfit group compared with the fit group (P = 0.006). Serum levels of inflammatory markers IL-6 and TNF-⍺ were 111.0% and 96.6% higher in the unfit group compared with the fit group (P = 0.04 and P = 0.02 respectively). No significant differences were detected between these two groups in skeletal muscle mitochondrial respiration capacity or thiol status at baseline. However, muscle nitrite was 58.7% lower in unfit patients compared to fit patients (P = 0.003), with no baseline differences in nitrate (Table 3).

**TABLE 3:** Baseline metabolic differences according fitness. A comparison of baseline serum oxidative/nitrosative markers, skeletal muscle mitochondrial respiratory capacity and redox-related tissue metabolites of unfit and fit patients.

		Unfit median (IQR)	Fit median (IQR)	Sig. P value
Baseline serum redox and inflammatory markers				
Adjusted TFT (µmoles/g protein)		4.21 (2.31)	4.94 (0.90)	0.30
FRAP (µM)		839.05 (726.17)	869.70 (563.23)	0.92
TBARS (µM)		7.50 (8.55)	4.32 (3.49)	0.11
HNE (ng/mL)		6.29 (5.74)	10.02 (5.76)	0.10
Isoprostanes (pg/mL)		251.31 (164.67)	228.90 (127.27)	0.41
cGMP (pg/mL)		154.49 (57.92)	85.03 (39.22)	0.006
Nitrite (µM)		0.17 (0.17)	0.17 (0.21)	0.45
Nitrate (µM)		33.25 (24.06)	33.60 (23.81)	0.92
RxNO (nM)		44.48 (90.01)	27.48 (12.59)	0.11
IL-6 (pg/mL)		4.77 (16.32)	2.26 (2.76)	0.04
TNF-a (pg/mL)		0.59 (0.56)	0.30 (0.25)	0.02
Baseline skeletal muscle mitochondrial respiratory capacity				
LEAK_FAO_ (pmoles^−1^ mg^−1^)		10.21 (8.64)	5.68 (4.41)	0.25
FAO_OXPHOS_ (pmoles^−1^ mg^−1^)		18.92 (15.57)	14.68 (6.94)	0.68
MOP_OXPHOS_ (pmoles^−1^ mg^−1^)		26.66 (20.44)	22.76 (5.05)	0.86
CI_OXPHOS_ (pmoles^−1^ mg^−1^)		28.55 (23.60)	26.57 (7.84)	0.95
MAX OXPHOS (pmoles^−1^ mg^−1^)		64.27 (43.73)	72.04 (28.23)	0.99
CI + II_ETS_ (pmoles^−1^ mg^−1^)		79.81 (14.14)	77.42 (30.86)	0.76
CII_ETS_ (pmoles^−1^ mg^−1^)		47.08 (23.00)	59.35 (33.15)	0.72
Baseline skeletal muscle metabolites free concentration				
Cysteine (nmoles/kg)		35.37 (15.45)	35.47 (37.85)	0.65
Homocysteine (nmoles/kg)		2.31 (5.69)	1.40 (0.69)	0.72
Sulfide (nmoles/kg)		1.22 (1.43)	1.99 (2.22)	0.27
GSH (mmoles/kg)		2.75 (3.30)	2.55 (0.82)	0.44
GSSG (nmoles/kg)		4.56 (47.48)	2.19 (5.13)	0.65
Baseline skeletal muscle metabolites total concentration				
Cysteine (nmoles/kg)		57.30 (33.14)	66.33 (30.15)	0.28
Homocysteine (nmoles/kg)		8.59 (14.48)	7.74 (3.71)	0.88
Sulfide (nmoles/kg)		62.11 (66.71)	51.26 (39.13)	0.80
GSH (mmoles/kg)		2.06 (1.99)	1.82 (0.86)	0.24
GSH:GSSG		1,159.00 (2069.10)	1,233.00 (1,344.50)	0.57
Skeletal muscle NO metabolites				
Nitrite (µmoles/kg)		11.95 (11.56)	16.83 (12.90)	0.19
Nitrate (µmoles/kg)		276.90 (1,028.30)	260.80 (203.30)	0.86

We then compared the magnitude of intraoperative *changes* (EoS-baseline) in the concentrations of circulating serum redox and inflammatory markers as well as skeletal muscle mitochondrial function and muscle metabolites between the fit and unfit groups to explore the effects of the surgery on these parameters. No differences were detected between groups in terms of intraoperative changes in serum TRC, lipid oxidation or nitrosative stress. The intraoperative reduction in serum TNF-⍺ levels in the fit group was 65.2% less than that reported in the unfit group (P = 0.03). No between-groups difference was detected in terms of changes in IL-6 (Supplementary Table S4).

The increase in intraoperative skeletal muscle mitochondrial LEAK_FAO_ was 200% higher in fit compared with unfit patients (P = 0.004). No other differences were observed between the two groups (Supplementary Table S4).

Peak VO_2_ was found to correlate with baseline blood measurements of TNF-⍺ (r = 0.491, P = 0.045, n = 17), baseline skeletal nitrite levels (r = 0.492, P = 0.045, n = 17) and with the degree of intraoperative changes in mitochondrial LEAK respiration (r = 0.730, P = 0.003, n = 14).

## Discussion

This sub-study aimed to improve our understanding of how major abdominal surgery affects mitochondrial respiration and redox status of skeletal muscle under conditions of systemically increased oxidative stress (results of main study reported elsewhere [13]), and whether these changes differ between fit and unfit individuals. The key findings include no intraoperative change in mitochondrial respiratory capacity, (FAO_OXPHOS_, CI_OXPHOS_ and MAX OXPHOS), suggesting that oxidative phosphorylation is preserved during this hyper-acute intraoperative period. However, an increase in respiration not coupled to oxidative phosphorylation (LEAK_FAO_ respiration) was detected by the end of surgery, along with an increase in skeletal muscle free cysteine levels. These changes were related to the patient’s cardiopulmonary fitness (as assessed by CPET): the intraoperative increase in LEAK respiration was greater in fit patients (and LEAK respiration also correlated positively with peak VO2?). In addition, baseline differences in inflammatory profile were detected in the fit and unfit groups, with plasma inflammatory markers (cGMP, IL-6 and TNF-⍺) found to be greater in less fit patients along with a lower level of skeletal muscle nitrite.

Our findings invite several plausible explanations that warrant further experimental investigation and, at this stage, are essentially hypothesis-generating. The increase in skeletal muscle LEAK_FAO_ respiration may be a sign of mitochondrial adaptation to rising systemic oxidative/nitrosative stress; explanations for a potential underlying mechanism are discussed below. The flexibility of skeletal muscle to increase LEAK_FAO_ respiration was also associated with increased physical fitness, where fitter patients demonstrated greater increases in LEAK_FAO_ intraoperatively than those with lower ATs. Moreover, baseline measurements of cGMP, IL-6 and TNF-⍺ were higher in unfit patients, who also had less nitrite in their skeletal muscle. The dichotomy in NO-related metabolites between skeletal muscle and circulating concentrations observed in the present study appears to be counter-intuitive insofar as the capacity to raise circulating nitrite concentrations (secondary to stimulation of endothelial NO synthase) in healthy individuals is typically a direct function of muscle mass and cardiorespiratory fitness [19]. However, whole-body regulation of these molecules in health is likely to differ from that in ill-health. Importantly, nitrite is not a passive oxidative breakdown product of NO but a reactive species and a signalling molecule in its own right [1, 20]. This includes the modulation of mitochondrial function [21] with complex interactions between endogenous NO production, dietary NOx intake and oral microbiome [22], cross-talk between nitrite and sulfide/persulfide-related (NO-independent) pathways [23, 24], oxygen-dependent tissue processing [25], and physical activity-dependent inter-organ exchange processes [20, 21]. Moreover, associated redox metabolism/signalling are subject to considerable alterations under inflammatory conditions [26, 27], making it difficult to predict the outcome of these interactions in the context of mitochondrial function and systemic inflammation. In any case, the differences in muscle nitrite observed in the present study may reflect either greater tissue utilization or leakage of nitrite from muscle into blood in frail patients. Why circulating cGMP levels were higher in less fit patients is similarly unclear but may be a reflection of higher iNOS expression and formation of peroxynitrite in either vasculature or circulating blood cells and linked to chronic inflammation.

Skeletal muscle free cysteine levels increased during the intraoperative period, with no change in glutathione concentrations. Since neither free cystine nor homocystine were detected (and levels of oxidized glutathione were vanishingly small), the differences between total and free thiol concentrations measured in skeletal muscle largely reflect the level of protein-bound thiols (i.e., the extent to which protein thiols are cysteinylated, homocysteinylated, glutathionylated and persulfidated). The differences in cysteine levels observed may be a sign of the muscle’s ability to elevate this sulfur-containing amino acid to maintain levels of glutathione in this tissue, in particular under conditions of increased oxidative stress. Since glutathione is of importance for much more than cellular antioxidant protection including, for example, mitochondrial function [24] it is tempting to speculate that the observed differences in cysteine concentrations in skeletal muscle before and after surgery may be linked to the ability of skeletal muscle to withstand major stress, thus reflecting biological resilience.

Regardless of these considerations, our findings suggest that cardiopulmonary fitness correlates more closely with intrinsic inflammatory levels than with oxidative/nitrosative stress in this cohort of patients with cancer. The raised baseline inflammatory state may also be a reflection of the cancer burden [28, 29], and the reduction in cardiopulmonary fitness may be the phenotypic expression of a state of functional loss and cachexia in cancer patients. In addition, raised systemic baseline IL-6 and reduced nitrate was found in patients who went on to develop severe morbidity [13]. These metabolic observations may reflect the complexity of metabolic regulation and response to surgical stress at the whole-body level [30] but are consistent with clinical observations that unfit patients are more likely to develop greater postoperative morbidity, which may be secondary to increased baseline inflammation, greater ROS production and a consecutively reduced bioavailability of NO (a powerful antioxidant) [31]. Skeletal muscle plays a vital role in inter-organ exchange of building blocks, redox regulation and metabolic flexibility. Sarcopenia, which is a hallmark of ageing and a measure of frailty, has been associated with chronic exposure to oxidative stress, inflammation and reduction in antioxidant capacity [32–34]. Raised levels of IL-6, which is independent of body composition, have been associated with reduced physical activity and increasing frailty [35, 36]. These findings may form part of the notion that fitter individuals have a lower baseline inflammatory state, which renders them to be more resilient and better able to adapt to stresses intraoperatively through mitochondrial protective mechanisms.

LEAK respiration is the dissipative component of mitochondrial respiration, which is independent of oxidative phosphorylation. The main contributor to this is proton leak, whereby a proportion of protons leak across the inner mitochondrial membrane through a route that is not coupled to formation of ATP, with this having both basal (i.e., unregulated) and inducible components [37]. The increased LEAK respiration observed here following surgery could therefore indicate increased proton leak, which might be mediated by protein-dependent or independent mechanisms. Mild uncoupling has been shown to lower ROS production in cellular models, at the expense of ATP production [38, 39]. In our study, the observation of increased LEAK respiration may therefore reflect an adaptive response to acute surgical stress at the muscular end-organ level.

In response to sepsis, upregulation of uncoupling protein 3 (UCP3) has been seen in mouse skeletal muscle [40, 41], whilst UCP3 expression is also elevated in human muscle in response to redox stress following shorter-term hypoxic exposure [16, 42]. Although the short timeframe of the intraoperative acute stress exposure in this study makes altered UCP expression unlikely, an alternative explanation could involve post-translational modification. For example, the glutathionylation of UCP3 has been shown to activate uncoupling [39]. Although we saw no change in overall GSH levels, our measure is reflective of skeletal muscle overall and not specifically the mitochondrial compartment. A UCP3-dependent mechanism might not, however, explain the greater increase in LEAK respiration seen in fit compared with less fit individuals in our study, since UCP3 expression (relative to mitochondrial content) is typically lower in the skeletal muscle of more trained individuals, in conjunction with enhanced mechanical efficiency [43]. Moreover, the physiological significance of any UCP3-mediated uncoupling is unclear, since in a mouse model of sepsis, survival rates were not different between wild-type and UCP3 knockout mice [41].

Instead, a non-UCP mediated mechanism might underpin our finding of a surgery-induced increase in LEAK; for example, in a hypoxic environment, NO can cause mild membrane depolarisation [44, 45]. Alternatively, the adenine nucleotide translocases (ANTs) have been proposed to be major fatty acid-inducible mediators of proton leak in many tissues, including skeletal muscle [46, 47]. Expression of the ANT1 isoform increased in human skeletal muscle in response to endurance training, and this was associated with greater sensitivity for fatty acid-mediated uncoupling [48]. This mechanism has been proposed to be protective against the development of insulin resistance in the event of fatty acid overload [48], but deserves further investigation in the contexts of redox stress, sepsis and surgical stress.

Comparison of the results of the present investigation to previous studies is challenging as the combinations of biological sampling sites, analytes and fitness measures using CPET in a surgical setting has not been undertaken before. Mitochondrial respiratory changes in skeletal muscle in a pig model after surgery have been investigated. Altered mitochondrial respiration was found when muscle fibres were biopsied 24 h apart and studied using HRR [8]. Increased fatty acid-mediated LEAK respiration was seen, which was directly comparable to our findings, along with evidence of no change in ADP-stimulated respiration with glutamate. Similarly, no reduction in ADP-stimulated respiration with pyruvate, was measured in our study. In a separate study, human skeletal muscle biopsies were taken before and after major abdominal surgery in patients with pancreatic cancer and benign disease. Muscle mitochondria were isolated and used to measure pyruvate dehydrogenase complex activity along with maximal ATP production, using bioluminescence. This study demonstrated reduced pyruvate dehydrogenase complex activity and a decreased rate of mitochondrial ATP production supported by palmitoyl-carnitine, and complex I and complex I&II-mediated substrates post-surgery [9]. Whilst we did not see a decreased capacity for O_2_ consumption supported by octanoyl-carnitine, or complex I and complex I&II substrates in combination in the oxidative phosphorylation state, the lower ATP production reported by Atkins and colleagues, might be explained by greater uncoupling as a result of proton leak (i.e., less ATP production per O_2_ consumed), and this would be in accordance with our findings and those of Hagve and colleagues in the porcine model.

Changes in tissue thiol levels have been observed previously, with skeletal muscle concentrations of cysteine and GSH remaining unchanged immediately after surgery, and GSH levels subsequently falling at 24 h [49], a timescale not measured in our study. Exercise and fitness-based studies, however, have demonstrated a positive correlation between levels of cGMP and fitness; physical training has been associated with increased circulating cGMP levels, for example, in hypertensive individuals [50]. The opposite was observed in this study, where less aerobically fit individuals demonstrated increased levels of cGMP. Evidence of increased inflammation and low CPET fitness has been previously reported in a study measuring preoperative CPET with neutrophil-leukocyte ratio as a measure of level of systemic inflammation, AT was found to be independently associated with neutrophil-leukocyte ratio [11]. In the context of our study of unfit patients with malignancy, multifactorial inflammatory mechanism may be contributing to the increased IL-6 and TNF-⍺ observed.

This study highlights several areas for potential further investigation, particularly in phenotyping patients to better understand the biology of resilience under acute surgical stress. This includes exploring the role of increased mitochondrial LEAK in preoperative fitness, the mechanisms underlying this process, and the function of skeletal muscle cysteine—specifically whether its presence serves a protective role during surgical stress. While this study focused on the acute intraoperative period, examining later physiological responses (e.g., on days three, five, and seven)—when postoperative complications commonly arise—could provide additional insights into protective metabolic responses and favorable characteristics for recovery.

### Study limitations

Our study was not initially designed to be powered to detect mitochondrial and metabolic changes in skeletal muscle. In addition, CPET was not part of the patients’ routine preoperative work-up. The voluntary performance of CPET could be a major confounder in this study, since the sub-group of patients investigated may have been physically fitter than the main study cohort. The lack of difference in oxygen consumption (in absolute terms) between the respiratory states during the perioperative period may partly be attributable to the short time-frame between biopsy extractions, since protein translational responses may not have occurred. Secondly, the vastus lateralis is remote from the site of surgical injury, so the tissue exposure to inflammatory changes may not have been as exaggerated as that experienced intra-abdominally or within the circulation.

## Conclusion

This sub-study offers novel insights into mitochondrial, redox, metabolic and inflammatory changes at a systemic and end-organ level before and acutely after surgery. Higher preoperative systemic inflammation levels, reduced tissue nitrite and blunted intraoperative LEAK respiration in unfit individuals may be hallmarks of inferior resilience, with overall preservation of mitochondrial respiratory capacity and GSH in skeletal muscle reflecting the whole-body ability to withstand major surgical stress. The exemplary approach taken in the present study may be suited to phenotype patients into subgroups of lesser or greater resilience or susceptibility to postoperative complications.

## Data Availability

The datasets presented in this study can be found in online repositories. The names of the repository/repositories and accession number(s) can be found in the article/Supplementary Material.
