# Supplementary material for: The effects of major abdominal surgery on skeletal muscle mitochondrial respiration in relation to systemic redox status and cardiopulmonary fitness
Source: Exp Biol Med (Maywood). 2025 Feb 21;250:10254. doi: 10.3389/ebm.2025.10254 (PMC11886423; doi:10.3389/ebm.2025.10254)
Supplement: Supplementary file 1 [file DataSheet1.docx]

# **Supplementary data**

to

**The effects of major surgery on mitochondrial respiration, redox metabolites, and the differences in these metabolic changes between cardiopulmonary fitness phenotypes**

Jia L Stevens Ph.D^1, 2^, Helen T McKenna Ph.D^3^, Helder Filipe BSc^2^, Magdalena Minnion^4^; Andrew J Murray Ph.D^5^; Martin Feelisch Ph.D^4^; Daniel S Martin Ph.D^3^

1. Department of Anaesthesia, Royal Sussex County Hospital, University Hospital Sussex NHS Foundation Trust, Eastern Road, Brighton, BN2 5BE, UK
2. University College London, Division of Surgery and Interventional Science, Royal Free Hospital, London NW3 2QG, UK
3. Peninsula Medical School, University of Plymouth, John Bull Building, Plymouth, Devon, PL6 8BU, UK.
4. Clinical & Experimental Sciences and Integrative Physiology and Critical Illness Group, Faculty of Medicine, Southampton General Hospital, University of Southampton, Southampton, UK
5. Department of Physiology, Development and Neuroscience, University of Cambridge, Cambridge, CB2 3EG, UK

## Supplementary Methods

## Anaesthetic and surgical techniques

The patients underwent induction of anaesthesia with the intravenous agents fentanyl and propofol, tracheal intubation using a non-depolarising muscle relaxant and maintenance of anaesthesia with a volatile anaesthetic. Neural-axial blockade was used for some patients in the form of epidural or spinal anaesthesia commenced prior to induction of anaesthesia. Patients also received a mixture of opioid and non-opioid analgesia for intra-operative pain relief. A central venous catheter was inserted into the internal jugular vein for venous sampling. The use of vasopressor / inotropic agents and intravenous fluids were commenced based on the physiological and hemodynamic changes observed intraoperatively. After induction of anaesthesia, patients underwent a laparotomy with a subcostal incision and midline extension. The surgical techniques included hepatic and pancreatic resections. The full spectrum of HPB surgery was included because these types of surgery carried theoretically the largest magnitude of surgical stress conducted at the study institution. This included a uniform L-shaped laparotomy, and anesthetic technique, with varying degrees of resection and vascular occlusion. Widening surgery to the HPB cohort and not just to one modality also aided recruitment of participants.

**Blood analysis**

The following markers were chosen on the basis of their relationship within the redox network: malondialdehyde (MDA), 4-hydroxynonenal (4-HNE), 8-iso-prostaglandin F_2⍺_ (8-isoprostanes), total free thiols (TFTs), ferric reducing ability of plasma (FRAP), cyclic guanosine monophosphate (cGMP), nitrite, nitrate and total nitroso-species (RxNO). Interleukin-6 (IL-6) and tumour necrosis factor alpha (TNF-⍺) were measured to evaluate inflammation. Pristine (first thaw from -80 ^o^C) serum or plasma samples were used throughout.

Lipid oxidation

Three markers were chosen to assess the extent of lipid oxidation. MDA was measured using the thiobarbituric acid reactive substance (TBARS) assay by colorimetry.^1^ The protein adducts of 4-HNE were measured using a competitive ELISA (Elabscience, E-EL-0128), and results were expressed relative to total protein.^2^ Total 8-isoprostanes were also measured using a competitive ELISA kit following alkaline hydrolysis (Cayman Chemical, 516360).

Total reducing capacity

Total reducing capacity was assessed by measuring both thiol-dependent (albumin) and metal-reactive antioxidants. Serum total free thiol levels were determined by a spectrophotometric method using dithionitrobenzoic acid, as previously described. ^3^ Free thiol concentrations were normalised to overall protein concentration, as serum proteins are the predominant source of thiols in serum/plasma.^4^ The ability of plasma to induce one-electron reduction of iron (FRAP assay) was also measured as previously described.^5^

Nitric oxide pathway

The involvement of the nitric oxide (NO) pathway was investigated by measurement of the oxidative end-products of NO metabolism (nitrite and nitrate), and downstream NO signalling (cGMP), as well as RxNO as a sensitive marker of nitrosative stress. Nitrite and nitrate concentrations were determined using high-performance liquid chromatography (ENO-20, EiCom);^6^ RxNO was quantified using gas-phase chemiluminescence (CLD88 am sp, Eco Medics).^7^ cGMP concentrations were determined using a commercially available enzyme immunoassay (R&D systems, KGE003).

Inflammatory markers

Serum IL-6 and TNF-α concentrations were measured using a commercially available high sensitivity ELISA kit (R&D systems, HS600C and HSTA00E respectively).

**Sample size calculation**

The nature of this sub-study was exploratory, to detect mitochondrial cellular changes during the perioperative period, from baseline to the end of surgery. Sample size was calculated using the ClinCalc sample size calculator (https://clincalc.com/stats/samplesize.aspx) with the parameters a=0.05, b=0.8. LEAK_FAO_ from a control group of patients who underwent hip surgery was measured in our laboratory and used for the sample size calculation.^8^ To account for attrition rates, a further 20% was added on to the calculated figure and rounded to the nearest ten (a sample size of 32 was obtained, and 38.4 if accounting for 20% attrition rate).

**Supplementary Figure 1.** Study flow diagram (CONSORT diagram)

76 patients refused to take part in the study

135 patients were eligible for recruitment

3 patients excluded from

the study

-1 unfit for surgery

-1 operated in the private

sector

-1 cancelled due to

ongoing chemotherapy

59 patients consented and enrolled. A sub-group of 26 patients were enrolled to perform CPET.

Enrollment

Preoperative

23 patients underwent CPET testing

37 patients underwent muscle biopsies and blood sample extraction at baseline and *EoS*

Sampling

37 paired muscle samples were used for metabolomics and 33 paired samples were used for HRR

Analysis

**Supplementary Table 1. SUIT protocol substrates and mitochondrial respiratory states**

| Substrate | mM^[[1]](#footnote-1)^ | Substrate / inhibitor characteristics | Respiratory state |
| --- | --- | --- | --- |
| Malate | 2 | CI substrate, unable to sustain respiration itself, >0.5 mM can inhibit CII-linked respiration. |  |
| Octanoyl-carnitine | 0.5 | Medium-chain FA, a substrate for FAO that feeds electrons directly to electron transfer flavoprotein and subsequently to coenzyme Q. Concentrations of FA were optimised to reach saturation without inducing inhibitory or uncoupling effects. Hence two titrations were added in this protocol. | LEAK_FAO_ |
| ADP | 5 | Impermeable to the cell membrane. A predefined and validated optimal concentration of saponin was used for permeabilisation. Magnesium was added as a co-factor for ATP synthase for the production of ATP. | FAO_OXPHOS_ |
| Pyruvate | 5 | Reconstitutes Kreb’s cycle. | MOP_OXPHOS_ |
| Glutamate | 10 | Maximal electron donation to CI. | CI_OXPHOS_ |
| Succinate | 50 | CII substrate; higher concentrations were required to overcome the inhibition of 2mM malate. | CI + CII_OXPHOS_ |
| ADP | 2.5 | Final addition was required to ensure that ATP synthase was saturated. | MAX OXPHOS |
| Cytochrome *c* | 0.01 | This was an outer mitochondrial membrane integrity test. During shuttling of electrons from CIII to CIV, if >15% JO_2_ was detected the result was excluded from analysis, as this was a sign of damage to the mitochondrial inner membrane. |  |
| FCCP |  | A protonophore that causes uncoupled respiration. Titrations of FCCP obtained the maximum mitochondrial respiration in the uncoupled state (electron transfer capacity). | CI + CII_ETS_ |
| Rotenone | 0.5(uM) | CI inhibitor. | CII_ETS_ |

**Supplementary Table 2. Intraoperative anaesthesia use**

| Intraoperative | N (%) |
| --- | --- |
| Anaesthesia |  |
| Induction |  |
| Propofol | 32 (94.1) |
| Dosing (mg) | 100 (100-172) median (IQR) |
| Antibiotics | 2 (5.9) |
| Opioid analgesia |  |
| Fentanyl | 34 (100) |
| Alfentanyl | 1 (2.9) |
| Remifentanyl | 1 (2.9) |
| Morphine | 0 (0) |
| Oxycodone | 0 (0) |
| Adjuncts |  |
| Midazolam | 24 (70.6) |
| Clonidine | 1 (2.9) |
| Lignocaine | 21 (61.8) |
| Ketamine | 19 (55.9) |
| Muscle relaxant |  |
| Atracurium | 3 (8.8) |
| Rocuronium | 30 (88.2) |
| Vecuronium | 1 (2.9) |
| Neuraxial blockade |  |
| Spinal | 17 (50) |
| Epidural | 9 (26.5) |
| None | 8 (23.5) |
| Maintenance |  |
| Desflurane | 31 (2.9) |
| Sevoflurane | 1 (2.9) |
| Isoflurane | 2 (5.9) |
| Nitrous oxide (used as a co-maintenance agent) | 1 (1.8) |
| Inotropes |  |
| Noradrenaline (mcg/kg/min) | 29 (85.3) |
| Minimum infusion range |  |
| Maximum infusion range |  |
| Other (Phenylepherine) | 0 2 (3.6) |
| Fluids |  |
| Total volume (L) | 5.8 (3.8-7.4) median (IQR) |

| Intraoperative events | N(%) |
| --- | --- |
| Major haemorrhage | 6 (17.6) |
| Blood loss (L) | 3 (1-5) median (IQR) |
| Peritoneal soiling | 1 (2.9) |
| Blood | 6 (17.6) |
| Number of RBC units transfused | 2.2 (1.1-7.5) median (IQR) |
| Fresh-Frozen plasma | 3 (8.8) |
| Platelets | 0 (0) |

**Supplementary Table 3. Major intraoperative events**

Supplementary Table 4. Baseline to end of surgery change in serum oxidative/nitrosative markers, skeletal muscle mitochondrial respiratory capacity and metabolomics compared across different anaerobic groups

|  | **AT £10**  **median**  **(IQR)** | **AT >10**  **median**  **(IQR)** |  | **Sig.** |
| --- | --- | --- | --- | --- |
| **Serum redox and inflammatory markers** | | | | |
| **Adjusted**  **TFT (µmoles/g protein)** | 0.61 (1.66) | 0.70 (1.39) | | 0.96 |
| **FRAP (µM)** | -36.06 (786.10) | -64.70  (414.0) | | 0.60 |
| **TBARS (µM)** | 0.15 (7.02) | 3.29 (6.16) | | 0.09 |
| **HNE adjusted**  **to protein (ng/ml)** | 0.03 (0.09) | 0.01 (0.04) | | 0.39 |
| **Isoprostanes**  **(pg/ml)** | -12.03 (110.63) | -13.81  (150.26) | | 0.53 |
| **cGMP (pg/ml)** | -45.47 (35.39) | -43.48 (47.71) | | 0.44 |
| **Nitrite (µM)** | -0.04 (0.15) | -0.04 (0.16) | | 0.77 |
| **Nitrate (µM)** | -7.65 (10.95) | -3.79 (13.53) | | 0.37 |
| **RxNO (nM)** | 37.59 (65.81) | 47.39 (60.46) | | 0.30 |
| **IL-6 (pg/ml)** | 270.70 (739.60) | 161.40  (148.30) | | 0.29 |
| **TNF-a (pg/ml)** | -0.09 (0.32) | -0.01 (0.05) | | 0.03* |
|  | | | | |
| **LEAK_FAO_** | -0.37 (0.88) | 3.40 (6.35) | | 0.001* |
| **FAO_OXPHOS_** | 0.66 (3.78) | 6.58 (15.65) | | 0.36 |
| **MOP_OXPHOS_** | 4.31 (9.86) | 3.53 (23.71) | | 0.70 |
| **CI_OXPHOS_** | 1.60 (8.50) | 13.76 (23.36) | | 0.19 |
| **MAX OXPHOS** | -1.85 (39.38) | 2.05 (27.68) | | 0.44 |
| **CI+II_ETS_** | -1.88 (43.34) | 3.03 (37.55) | | 0.70 |
| **CII_ETS_** | 1.56 (7.95) | 5.75 (11.32) | | 0.66 |
|  | | | | |
| **Cysteine (nmoles/kg)** | 2.14 (31.81) | 24.93 (76.46) | | 0.33 |
| **Homocysteine (nmoles/kg)** | -0.27 (4.77) | -0.16 (1.30) | | 0.27 |
| **Sulfide (nmoles/kg)** | -0.42 (1.86) | 0.33 (1.75) | | 0.51 |
| **GSH (mmoles/kg)** | -0.36 (2.18) | -0.35 (1.72) | | 0.90 |
| **GSSG (nmoles/kg)** | 1.35 (44.63) | -0.02 (5.77) | | 0.38 |
| **Skeletal muscle metabolomics total concentration** |  |  | |  |
| **Cysteine (nmoles/kg)** | 10.42 (20.27) | 32.09 (135.60) | | 0.44 |
| **Homocysteine (nmoles/kg)** | -0.65 (14.91) | 0.58 (5.53) | | 0.65 |
| **Sulfide (nmoles/kg)** | -11.61 (124.34) | 6.95 (52.54) | | 0.88 |
| **GSH (mmoles/kg)** | -0.67 (1.46) | -0.29 (0.82) | | 0.57 |
| **GSH:GSSG** | -256.60 (1887.80) | -203.50 (2057.40) | | 0.72 |
| **Skeletal muscle** |  |  | |  |
| **Nitrite (μmoles/kg)** | -4.44 (11.64) | 5.01 (26.93) | | 0.51 |
| **Nitrate (μmoles/kg)** | -32.73 (5409.80) | -28.45  (320.20) | | 0.65 |

1. Feldman, E. Thiobarbituric Acid Reactive Substances (TBARS) Assay. *Diabetic Complications Consortium* (2019).

2. Spickett, C. M. The lipid peroxidation product 4-hydroxy-2-nonenal: advances in chemistry and analysis. *Redox biology* **1**, 145-152 (2013).

3. Koning, A. M. et al. Serum free thiols in chronic heart failure. *Pharmacological Research* **111**, 452-458 (2016).

4. Turell, L., Radi, R. & Alvarez, B. The thiol pool in human plasma: the central contribution of albumin to redox processes. *Free Radical Biology and Medicine* **65**, 244-253 (2013).

5. Benzie, I. F. F. & Strain, J. J. The ferric reducing ability of plasma (FRAP) as a measure of “antioxidant power”: the FRAP assay. *Analytical biochemistry* **239**, 70-76 (1996).

6. Horscroft, J. A. et al. Metabolic basis to Sherpa altitude adaptation. *Proceedings of the National Academy of Sciences of the United States of America* **114**, 6382-6387 (2017).

7. Feelisch, M. et al. Concomitant S‐, N‐, and heme‐nitros (yl) ation in biological tissues and fluids: implications for the fate of NO in vivo. *The FASEB Journal* **16**, 1775-1785 (2002).

8. McKenna, H. T. et al. Divergent trajectories of cellular bioenergetics, intermediary metabolism and systemic redox status in survivors and non-survivors of critical illness. *Redox Biology* 101907 (2021).

1. Concentration added per titration [↑](#footnote-ref-1)
